# Heterozygous pathogenic *STT3A* variation leads to dominant congenital glycosylation disorders and functional validation in zebrafish

**DOI:** 10.1186/s13023-025-03557-y

**Published:** 2025-01-31

**Authors:** Linxue Meng, Zhixu Fang, Li Jiang, Yinglan Zheng, Siqi Hong, Yu Deng, Lingling Xie

**Affiliations:** 1 Department of Neurology, Children’s Hospital of Chongqing Medical University, No. 136, Zhongshan Er Road, Yuzhong District, Chongqing, 400014 People’s Republic of China; 2 Department of Radiology, Children’s Hospital of Chongqing Medical University, Chongqing, People’s Republic of China; 3 National Clinical Research Center for Child Health and Disorders, Chongqing, People’s Republic of China; 4 China International Science and Technology Cooperation Base of Child Development and Critical Disorders, Chongqing, People’s Republic of China; 5 Ministry of Education Key Laboratory of Child Development and Disorders, Chongqing, People’s Republic of China; 6 Chongqing Key Laboratory of Pediatrics, Chongqing, People’s Republic of China

**Keywords:** *STT3A* gene, Congenital glycosylation disorders, Dominant inheritance, Zebrafish, Phenotype

## Abstract

**Background:**

Congenital disorders of glycosylation are a rare group of disorders characterized by impaired glycosylation, wherein *STT3A* encodes the catalytic subunit of the oligosaccharyltransferase complex, which is crucial for protein N-glycosylation. Previous studies have reported that *STT3A*-CDG is caused by autosomal recessive inheritance. However, in this study, we propose that *STT3A*-CDG can be pathogenic through autosomal dominant inheritance.

**Methods:**

The variant was identified via trio whole-exome sequencing. We constructed wild-type and variant plasmids, transfected them into HEK293T cells and detected the expression levels of the STT3A protein. We performed CRISPR-Cas9 to establish heterozygous knockdown zebrafish to validate the functional implications of autosomal dominant inheritance of *STT3A* in pathogenesis.

**Results:**

The patient presented with developmental delay, distinctive facial features, short stature, and abnormal discharges. The heterozygous pathogenic missense variant (NM_001278503.2: c.499G > T, NP_001265432.1:p. Asp167Tyr) was identified, and the Western blot results revealed a significant decrease in protein levels. Heterozygous knockdown zebrafish exhibit phenotypes similar to those of patients, including craniofacial dysmorphology (increased eye distance, increased Basihyal’s length, increased Ceratohyal’s angle), skeletal abnormalities (reduced number of mineralized bones), developmental delay (reduced adaptability under light‒dark stimuli suggesting abnormal locomotion, orientation, and social behavior), and electrophysiological abnormalities.

**Conclusion:**

We report a proband with a dominant congenital glycosylation disorder caused by heterozygous pathogenic *STT3A* variation, which is a new inheritance pattern of *STT3A*. Our report expands the known phenotype of dominant *STT3A*-CDGs. Furthermore, we provide in vivo validation through the establishment of a heterozygous knockdown zebrafish model for *stt3a* and strengthened the compelling evidence for dominant *STT3A*-related pathogenesis.

## Introduction

Congenital glycosylation disorders (CGDs) are a group of rare diseases caused by defects in the synthesis of N- or O-linked glycans of glycoproteins or defects in their attachment to the polypeptide chains of proteins [1–4]. CGD typically results in a wide range of multisystem clinical manifestations [5, 6]. With the advancement of genetic testing technologies, an increasing number of pathogenic genes related to CDG have been discovered. CDGs that impact the biosynthesis of lipid-linked oligosaccharide (LLO) in the endoplasmic reticulum (ER) or the transfer of N-glycan to recipient precursor glycoproteins are termed type I CDGs [7]. The proper functioning of the oligosaccharyltransferase (OST) complex is crucial for ensuring the correct glycosylation of proteins, as it is involved in transferring oligosaccharide chains onto proteins within the endoplasmic reticulum. OST is a polymeric protein complex consisting of STT3A (OST-A complex) or STT3B (OST-B complex) as catalytic subunits and a series of auxiliary proteins [8, 9]. OST-A catalyzes cotranslational N-linked glycosylation for most proteins upon entry into the ER, whereas OST-B performs posttranslational glycosylation at sites missed by OST-A, such as those located at the C-terminus of glycoproteins [10, 11].

Recently, pathogenic variants in various OST subunits have been demonstrated to cause autosomal-recessive or X-linked type I CDGs, such as *TUSC3*-CDG (MIM: 611,093) [12], *MAGT1*-CDG (MIM: 301,031) [13], *STT3A*-CDG (MIM: 615,596), and *STT3B*-CDG (MIM: 615,597) [14]. CDG caused by mutations in *STT3A*, also known as *STT3A*-CDG, was first reported in a Pakistani family in 2013 [14]. In this family, two affected siblings primarily presented with microcephaly, developmental delay, epileptic seizures, and hypotonia, both of whom carried the homozygous variant c.1877C > T. In 2017, Arunabha Ghosh et al. reported five independent individuals with CDGs carrying the same homozygous variant [6]. Therefore, to date, *STT3A*-CDG is still widely considered to be autosomal recessive in inheritance.

In this study, we assessed the clinical and genetic characteristics of the first Asian case of dominantly inherited *STT3A*-CDG, and we established heterozygous knockdown zebrafish for in vivo experiments to verify the potential of *STT3A* heterozygous variants causing dominant inherited pathogenicity.

## Materials and methods

### Patient

The patient was registered at the Children’s Hospital of Chongqing Medical University (CHCMU), the largest pediatric medical center in Southwest China. We collected demographic information, clinical data, biochemical data, imaging examination results, and genetic testing data for this patient.

### Next-generation sequencing (NGS)

Targeted NGS was performed following previously reported experimental procedures [15]. The average sequencing depth was 123.810. The sequencing result was aligned to the Genome Reference Consortium *Homo sapiens* (human) genome assembly GRCh37 (GRCh37/hg19), and all variants with a minor allele frequency of ≤ 0.05 in a public database (ExAC, gnomAD, 1000 Genomes) were selected for pathogenicity evaluation according to the American College of Medical Genetics and Genomics (ACMG) criteria. The *STT3A* variant was compared with the established human *STT3A* sequence (NM_001278503.2). Sanger sequencing was performed to validate the variants identified via next-generation sequencing (NGS) and for segregation analysis. Amino acid sequence alignment was performed via Genedoc software (https://github.com/karlnicholas/GeneDoc) for conservation analysis. PROVEAN (http://provean.jcvi.org/index.php), SIFT (https://sift.bii.a-star.edu.sg/), Polyphen-2 (http://genetics.bwh.harvard.edu/pph2/), and MutationTaster (https://www.mutationtaster.org/) were used to predict the pathogenicity of the variants. Protein modeling was conducted to anticipate the impact of missense variants on molecular structure, which was achieved via SWISS-MODEL [16]. The PyMOL software subsequently facilitated three-dimensional protein structure visualization and analysis.

### Plasmid constructs for generating the wild-type (WT) and *STT3A* variants

*STT3A* WT pIRES2-EGFP-FLAG (NM_001278503) and *STT3A* VARIANT pIRES2-EGFP-FLAG (NM_001278503.2) plasmids were purchased from the YouBao Company in Changsha. Then, we performed Sanger sequencing to check the full sequence of the coding region for each plasmid and ensure that there was no secondary mutation.

### Cell culture, transfection, and western blotting

Human embryonic kidney 293 T (HEK293T) cells (RRID: CVCL_0063) were cultured in Dulbecco’s modified Eagle’s medium (DMEM) supplemented with 10% fetal bovine serum. The cells were maintained in a controlled humidified environment at 37 °C in the presence of 5% CO_2_. When cells grow to 70–80% confluence, plasmid transfection can be performed. Prior to transfection, the medium of the cells was fully replenished with fresh medium, followed by the addition of the plasmid mixture (2.5 µg of WT or VARIANT plasmid in 4 µl of Lipo8000 and 125 µl of DMEM). Protein extraction was conducted 48 h after transfection.

We used a total protein extraction kit (BestBio) to extract total protein. The protein concentration was assessed via a Bio‐Rad protein assay kit (Bio‐Rad Laboratories). Samples containing 20 μg of protein were separated via 12% SDS polyacrylamide gel electrophoresis and transferred to polyvinylidene difluoride membranes (0.22 μm, Millipore). The membrane was blocked for 1 h at room temperature in PBS containing 5% bovine serum albumin and further incubated with specific primary detection antibodies (anti‐DYKDDDDK tag, mouse monoclonal antibody (Proteintech; 1:5000)) overnight at 4 °C. Subsequently, peroxidase–conjugated secondary antibodies were applied to the same membranes for another 1 h at room temperature. The protein bands were visualized via Clarity Western electrochemiluminescence (ECL) substrate (Bio‐Rad) and analyzed via densitometry using Image Lab software (NIH).

### Zebrafish maintenance

Adult zebrafish of the Tg [huc:GFP] (used for behavioral/microscopic morphology/electrophysiology experiments) and Tg [sox10:GFP] (used for observation of mandibular cartilage morphology/bone staining experiments) [17] strains were maintained in a circulating water environment at 28.5 ± 0.5 °C with a light/dark cycle of 14 h light/10 h dark [18]. The fish were fed twice daily with a diet consisting of brine shrimp and pellet fish. The zebrafish embryos used in the experiments were generated through the crossbreeding of adult fish, with parental zebrafish placed in hybrid breeding tanks at a 1:1 or 1:2 male-to-female ratio. The resulting embryos were raised in E3 media (0.03% sea salt, 0.00014% methylene blue in reverse osmosis water) and kept in a 28.5 ± 0.5 °C incubator (constant temperature). Dead embryos were removed daily during cultivation, and 30% of the culture medium was replaced regularly.

### Gene editing of zebrafish

The orthologous gene of *STT3A* in the zebrafish genome was identified as *stt3a* (ENSDARG00000104953) via the DIOPT Ortholog Finder (https://www.flyrnai.org/cgi-bin/DRSC_orthologs.pl). Single guide RNA (sgRNA) targets were pinpointed via the CHOPCHOP (version 3) online tool (https://chopchop.cbu.uib.no) [19] and subsequently procured from GenScript (Nanjing, China). The following four sgRNAs were designed for the targeted gene: sgRNA1, CCTCATTCCTCTGCACGTGCTGG; sgRNA2, GTTCTCTCATCGCATCTATGTGG; sgRNA3, CTACTGCACAGTCTACTGCCTGG; and sgRNA4, TACTGCACAGTCTACTGCCTGGG. To validate the editing efficiency of the four sgRNAs, we injected 1 nl of CRISPR complexes containing one sgRNA (at a concentration of 90 ng/µL each) or Cas9 protein (at a concentration of 250 ng/µL). Five embryos were taken from each group to prepare genomic DNA templates twenty-four hours after injection, which were then subjected to Sanger sequencing to verify the mutation effect. Finally, we used the TIDE algorithm (https://tide.nki.nl/) to confirm the editing efficiency.

### Observation of mandibular cartilage morphology in zebrafish

Eight-day postfertilization (dpf) Tg[sox10:GFP] zebrafish larvae were selected for observation of mandibular cartilage morphology. The experiment consisted of a Cas9 control group and a *stt3a* crispant group. Zebrafish larvae were placed belly up and fixed onto slides via 2% low-melting agarose gel.

### Bone staining in zebrafish

Nine dpf zebrafish larvae from both the Cas9 control group and the *stt3a* crispant group were selected for bone staining. The larval samples were fixed overnight at room temperature in 4% paraformaldehyde, washed twice with 1 × PBS, and stained with 0.1% Alizarin Red at room temperature for 30 min. The samples were subsequently bleached with 1.5% H_2_O_2_/0.25% KOH solution until decolorization, followed by another two washes with 1 × PBS. The samples were subsequently decolorized for 2 h in a mixture of 20% glycerol/0.25% KOH. Finally, the samples were stored in a 50% glycerol/0.25% KOH mixture and imaged under a dissecting microscope.

### Behavioral testing in zebrafish

The samples were divided into a Cas9 control group and a *stt3a* crispant group, with both groups undergoing simultaneous testing in the same 96-well plate. The testing procedures are as follows:

1. Spontaneous behavior: Zebrafish larvae at 5 dpf were selected and placed at a rate of 1 larva per well in a 96-well plate. After placing the 96-well plate into the DanioVision (Noldus) zebrafish behavioral tracking system, the larvae were allowed to adapt to the dark environment for 30 min before spontaneous behavior tests were conducted.
2. Light‒dark stimulation: Following the spontaneous behavior test, the light‒dark stimulation test was conducted continuously. Zebrafish larvae were subjected to alternating light‒dark stimuli for a total of four cycles, with each cycle consisting of 5 min of darkness followed by 5 min of brightness (light intensity set at 100% system value).

### Electrophysiology in zebrafish

Zebrafish larvae aged 5–6 dpf were selected for electrophysiological testing. Single zebrafish larvae were placed into sample wells, the excess original culture medium was removed, and a drop of E3 solution was added. The samples were then placed in a 4 °C refrigerator for 1 min and removed, after which the zebrafish were immobilized on the dorsal side with 2% low-melting agarose gel. After the agarose gel solidified, the samples were transferred to the experimental recording chamber, and E3 culture medium was added to cover the agarose and reference electrode. Local field potentials (LFPs) from the brain were recorded via glass microelectrodes inserted through the cranial vault. The glass microelectrodes were filled with 2 M sodium chloride solution. Electrical signals were recorded via a high-impedance electrophysiology amplifier (A-M Systems) and digitized via a data acquisition system (Measurement Computing) with a sampling frequency of 10 kHz and a filter set at 1 Hz to 5 kHz.

### Statistical analysis

Statistical analysis and graphing of the data were performed via GraphPad Prism 8. The normally distributed data are expressed as the mean ± standard error of the mean (SEM). The data that did not satisfy a normal distribution are presented as the median (interquartile range). The unpaired t test was used to compare two variable groups. The chi-square test was used for epileptiform event incidence analysis. A significance threshold of *p* < 0.05 was used (*p < 0.05, **p < 0.01, ***p < 0.001).

## Results

### Clinical features of the proband

The proband is from Yunnan, China, and is of Han ethnicity. She presented to our hospital primarily because of developmental delay. She was born at full term with a birth weight of 2.15 kg and a length of 48 cm. Newborn screening did not reveal any abnormalities. There were no perinatal risk factors, such as birth asphyxia, intrauterine growth retardation, meconium staining of amniotic fluid, or placental abruption. She has a brother and a sister, both of whom are currently healthy with no developmental abnormalities or distinctive facial features. Her parents are nonconsanguineous, and there is no history of similar presentations in the family.

Since early childhood, the patient has experienced significant delays in gross motor development. At 5 months, she could hold her neck up; at 11 months, she could sit independently; and at 2 years, she started walking independently. Currently (5 years and 7 months), she can run up and down the stairs but cannot hop on one foot. In addition to motor development, there are delays in her intellectual development. She started producing vowel sounds at 6 months, said "baba, mama" at 15 months, began using words at 2 years, formed short sentences at 3 years, and she can now (5 years and 7 months) recite simple ancient poems but does not construct logical narratives.

**Fig. 1:**
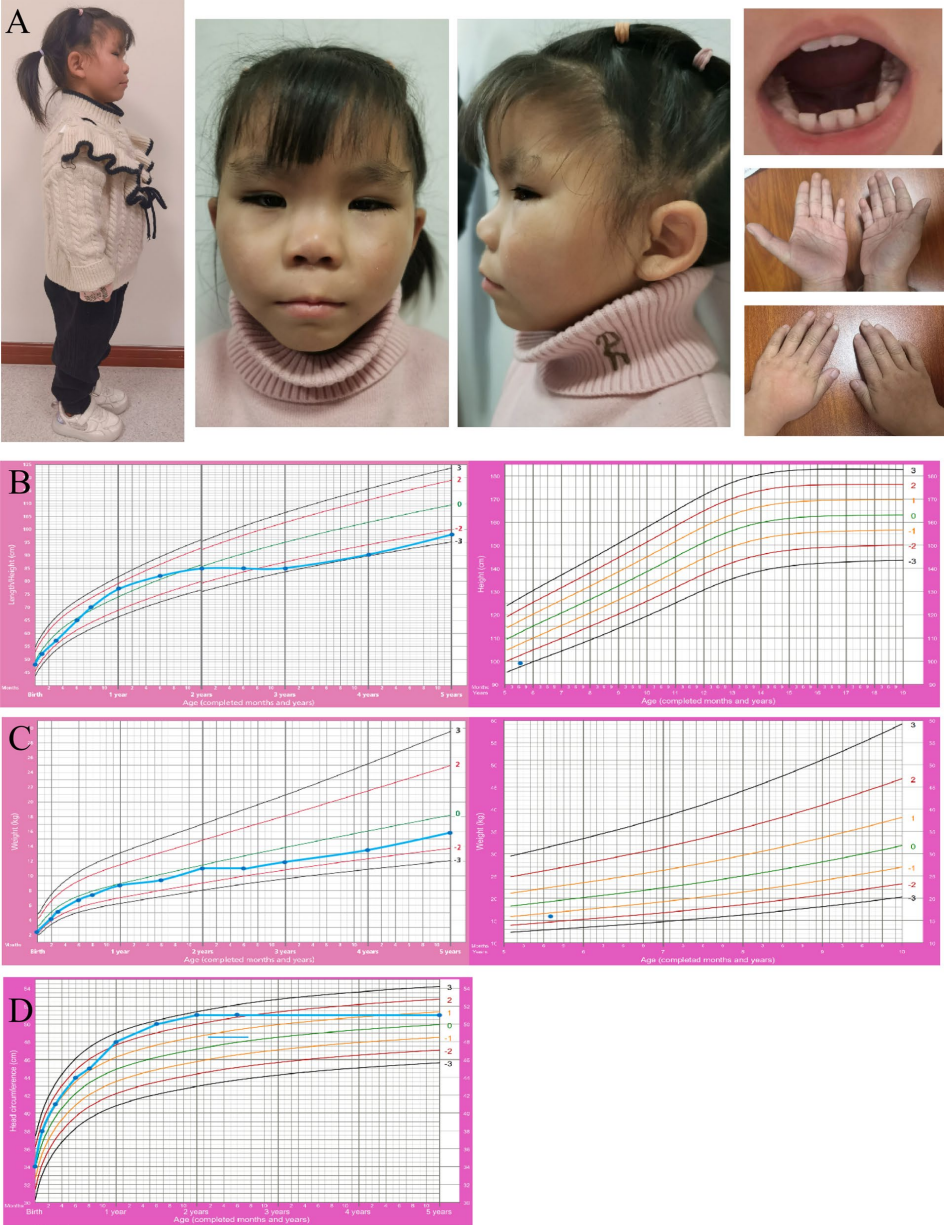
Clinical images of the proband. **A** Distinct facial features of the proband, including midfacial hypoplasia, prominent forehead, long face, almond-shaped eyes, short nose, long philtrum, thin upper lip, large and protruding ears, and sparse teeth, without obvious short and stubby fingers, deformities in the limbs or joint contractures. **B** Growth curve chart for the proband’s height/length. **C** Growth curve chart for the proband’s weight. **D** Growth curve chart for the proband’s head circumference

A detailed physical examination revealed distinct facial features, including midfacial hypoplasia, a prominent forehead, a long face, almond-shaped eyes, a short nose, a long philtrum, a thin upper lip, large and protruding ears, and sparse teeth. She had no obvious short or stubby fingers and no deformities in the limbs or joint contractures (Fig. 1A). We traced her growth and development from birth and plotted growth charts (height, weight, and head circumference) (Fig. 1B, D). Her height was within the normal range during infancy, and her growth rate was satisfactory. However, after the age of one, her height growth gradually slowed and gradually fell behind that of 95% of her peers. Currently, her height is 98.6 cm (< X-2SD), which indicates that she has short stature. The proband’s weight has consistently remained within the normal range, and her weight is now 16 kg (< X-1SD, > X-2SD). Although the head circumference of the proband exceeded that of 95% of her peers at the age of one, the rate of head circumference growth gradually decreased as development progressed, and her head circumference is now 51 cm (< X + 1SD), which is within the normal range. Neurological examination revealed no significant abnormalities.

### Auxiliary examination

**Fig. 2:**
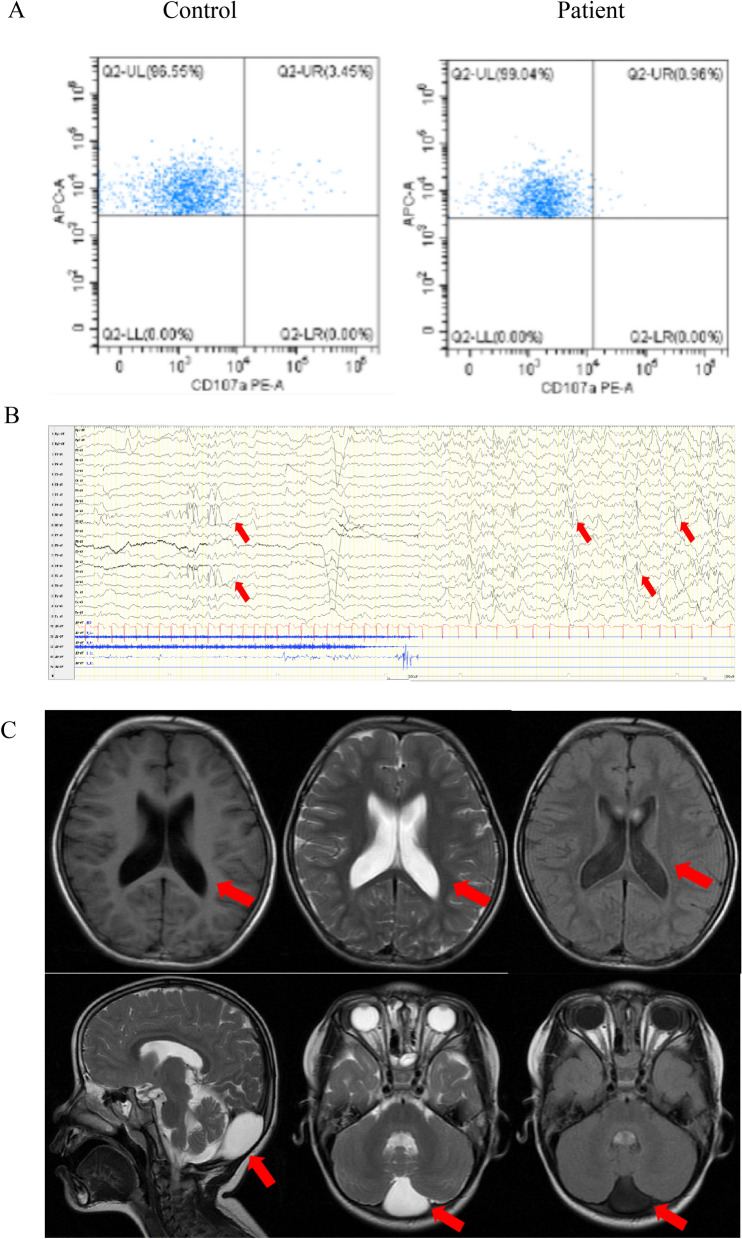

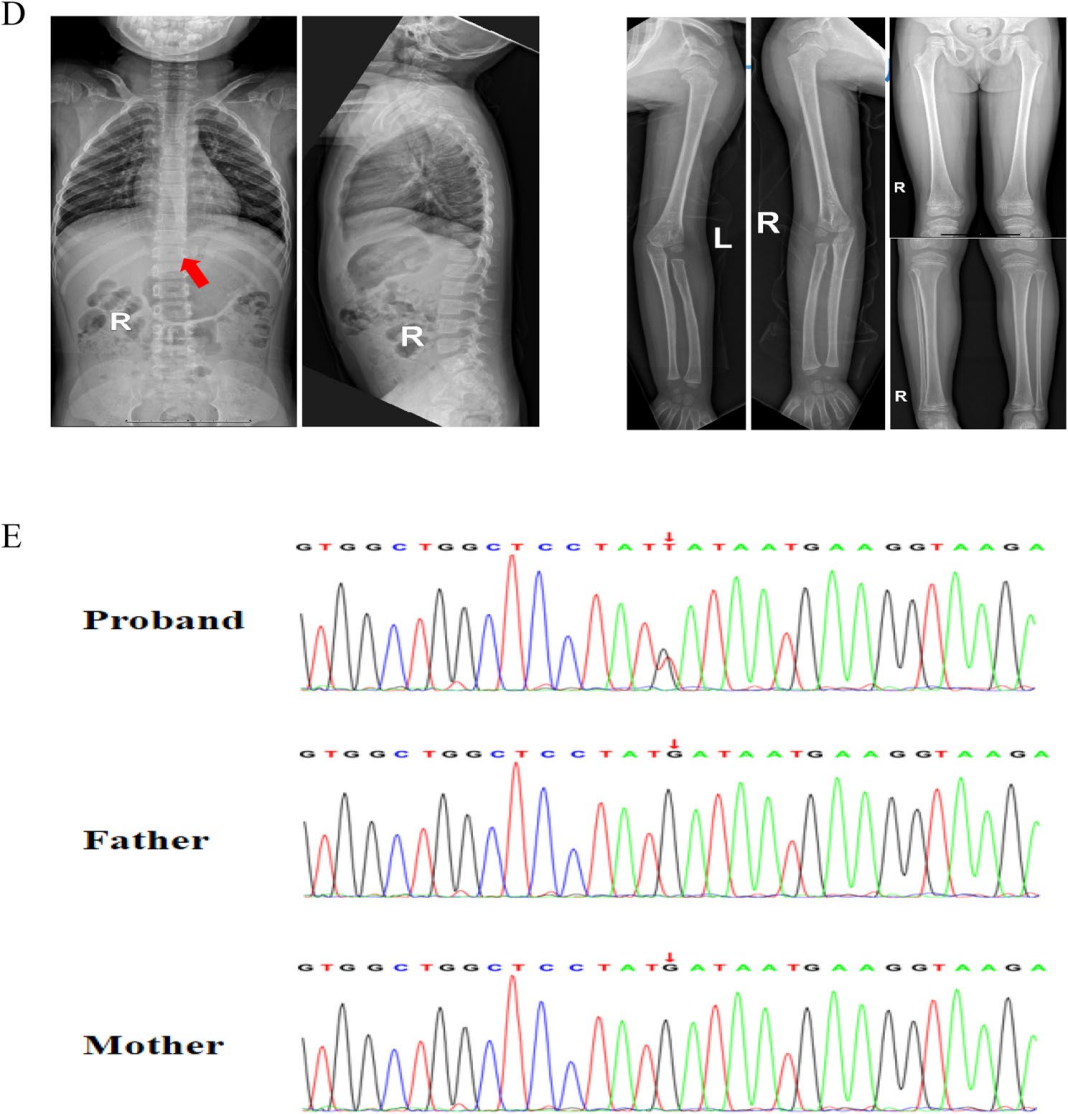
Auxiliary examination of the proband. **A** The peripheral blood flow cytometry results of the proband indicated a significant decrease in CD107a in the NK cells. **B** Video electroencephalogram revealed that the proband had abnormal discharges. In the awake state, moderate theta activity was detected at 4–7 Hz in the background without an occipital dominant rhythm. During wakefulness and sleep, spike-slow wave or slow wave bursts were observed in the left parietal and posterior temporal regions, which spread diffusely. **C** Brain magnetic resonance imaging (MRI) revealed an arachnoid cyst in the occipital region, along with bilateral enlarged lateral ventricles. **D** Spinal X-rays showing mild scoliosis with a Cobb angle of 4°, as indicated by the arrows. Limb X-rays revealed no abnormalities. **E** The Sanger sequencing of trios

The patient's comprehensive blood biochemistry examination (including complete blood count, liver and kidney function tests, thyroid function tests, insulin levels, growth factor levels, and vitamin levels) and metabolic screening (including blood ammonia, blood lactate, blood glucose, blood amino acid tandem mass spectrometry, and urine organic acid gas chromatography) revealed no abnormalities. However, the peripheral blood flow cytometry results of the proband indicated a significant decrease in CD107a in NK cells, the expression of which requires OST-A to catalyze N-glycosylation [20] (Fig. 2A). The patient underwent further chromosome examination, and the results revealed a chromosomal karyotype of 46,XX, which is consistent with her biological sex. At the age of 4 years and 3 months, she underwent Wechsler Intelligence Scale for Children (WISC) testing, which yielded a language IQ score of 57, a performance IQ score of 55, and a full-scale IQ score of 53, indicating that she has intellectual disability. Ophthalmological examination revealed refractive errors in both eyes, with a prescription of −4.2 diopters for the left eye and −4.4 diopters for the right eye, with no abnormalities observed in the fundus.

We conducted neurophysiological and imaging tests on the patient. Despite the absence of clinical discharges, video electroencephalogram (VEEG) results indicated that the patient had abnormal discharges (Fig. 2B). Specifically, in the awake state, there was moderate theta activity at 4–7 Hz in the background, without an occipital dominant rhythm; during wakefulness and sleep, spike-slow wave or slow wave bursts were observed in the left parietal and posterior temporal regions, which spread diffusely. Brain magnetic resonance imaging (MRI) revealed an arachnoid cyst in the occipital region, along with bilateral enlarged lateral ventricles (Fig. 2C). Additionally, spinal X-rays revealed mild scoliosis with a Cobb angle of 4°, with no apparent abnormalities in the long bones of the limbs (Fig. 2D).

### Identification of *STT3A* variants

**Fig. 3:**
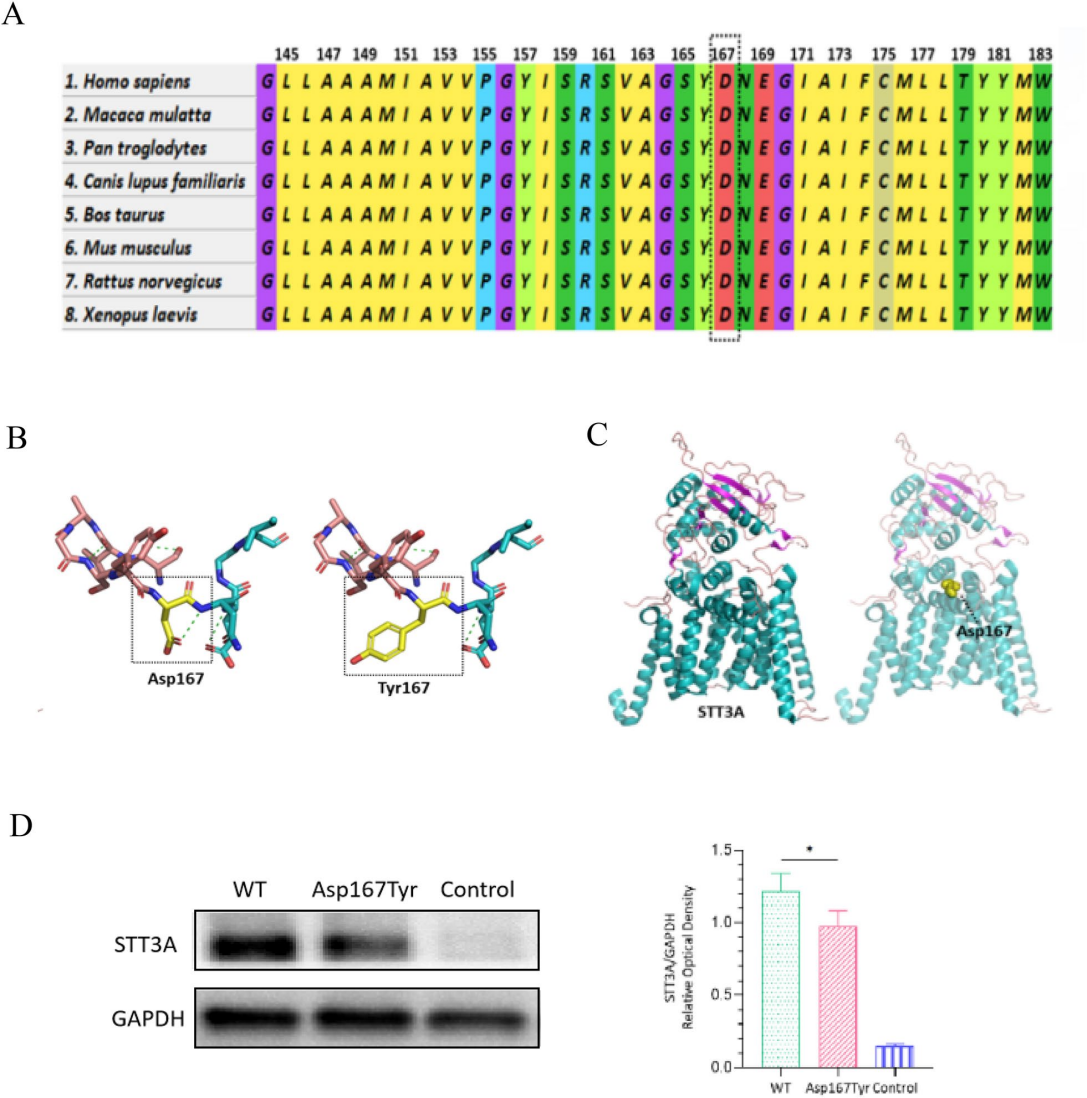
Characteristics of the variant and protein levels of STT3A. **A** Conservation analysis of this variant, which is conserved across multiple species. **B** Molecular modeling of the variant indicated a reduction in hydrogen bonds around the residue. **C** Molecular modeling of STT3A showing that the heterozygous missense variant is located within the binding site. **D** Western blot analysis of whole-cell protein extracts from transfected HEK293T cells. Compared with that in the WT group, the STT3A expression level in the Asp167Tyr group was significantly lower

We performed trio-WES on the patient and their family members. Initially, the test results did not reveal any gene variants consistent with familial segregation or inheritance. However, upon reanalysis of the sequencing data, we identified a de novo variant in *STT3A* carried by the patient. Sanger sequencing of this variant is shown in Fig. 2E. This is a heterozygous missense variant (NM_001278503.2: c.499G > T, NP_001265432.1:p. Asp167Tyr) and was classified as pathogenic (PS2 + PM1 + P2 + PP2 + PP3) according to the ACMG criteria. The conservation analysis suggested that this variant is conserved across multiple species (Fig. 3A). The variant has not been found in the normal population and is predicted to have strong pathogenicity via multiple online prediction tools (Table 1). Finally, we performed homology modeling of STT3A and constructed the mutated amino acid residue. We found that after the mutation of Asp to Tyr at position 167, there was a reduction in the number of hydrogen bonds around the residue, leading to a change in the protein structure (Fig. 3B). Additionally, this variant site is located within the binding site (Fig. 3C) and near the active site of STT3A [21]. The above evidence led us to suspect that this patient may be pathogenic for this variant. However, *STT3A* has been previously reported to be pathogenic with biallelic mutations. Therefore, we conducted follow-up cell and zebrafish experiments to verify the pathogenicity of the heterozygous state.

**Table 1:** Genetic features of the *STT3A* variant

Position	cDNA change (NM_001278503)	Protein change	Inherit	MAF	MAF-EAS	Provean	Polyphen2	Mutationtaster	SIFT
chr11:125474133	C.449 G > T	p.Asp167Tyr	*de nove*	–	–	Deleterious (−8.58)	Porobably damaging (1.0)	Disease_causing (1.0)	Damaging (0.0)

MAF, minor allele frequency from Genome Aggregation Database; MAF-EAS, minor allele frequency from Genome Aggregation Database–East Asia population

### Pathogenicity validation of the variant in vitro

We transfected the *STT3A* WT plasmid and p. Asp167Tyr VARIANT plasmid into HEK293T cells to assess the STT3A protein expression levels. The results revealed that the STT3A expression level in the p.Asp167Tyr group was significantly lower than that in the WT group (unpaired *t* test, *p* value < 0.05) (Fig. 3D). Thus, we speculate that this variant may be pathogenic and could lead to partial loss of OST-A function by reducing STT3A protein expression stability. However, further in vivo validation is needed.

### Zebrafish validation

**Fig. 4:**
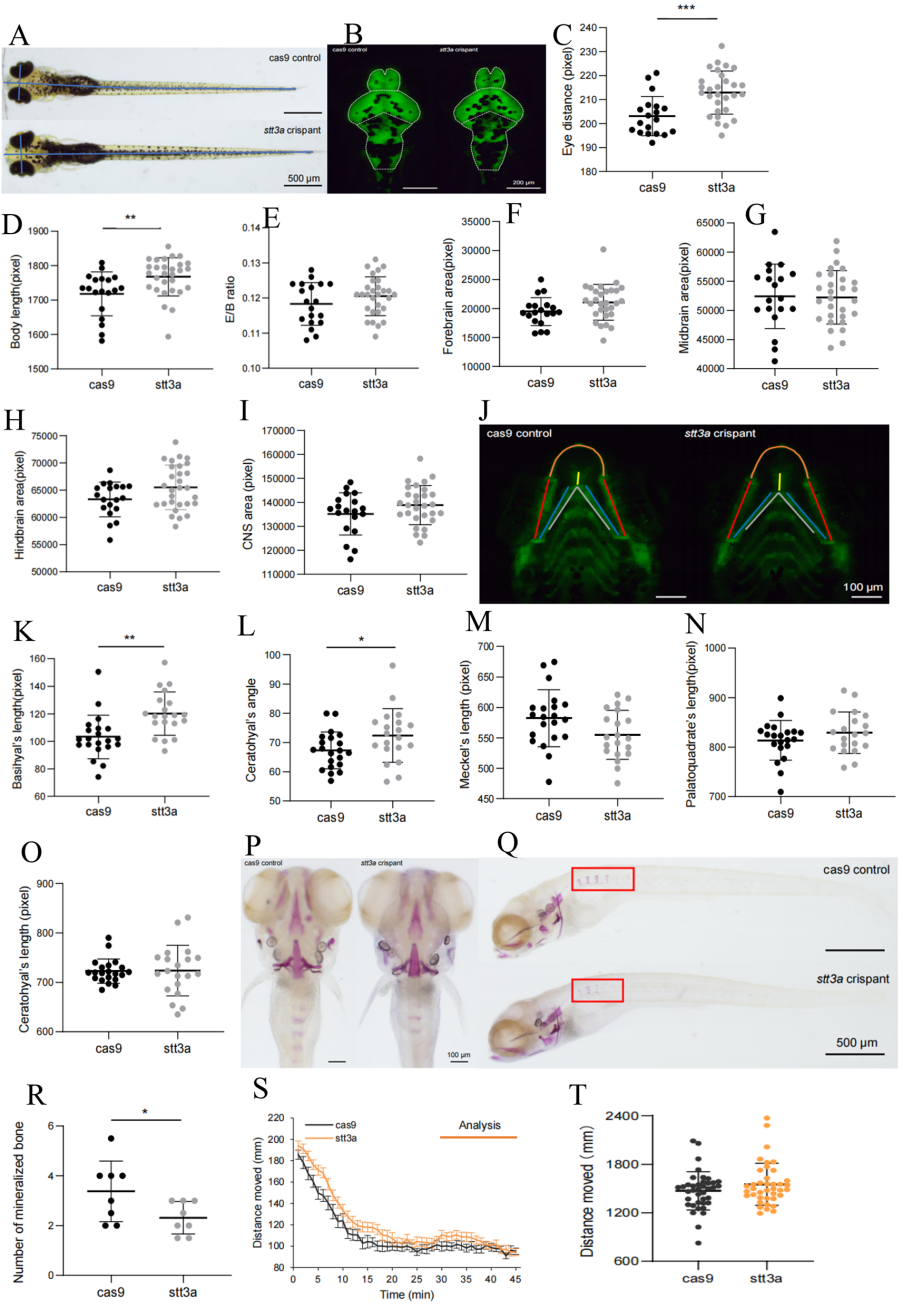

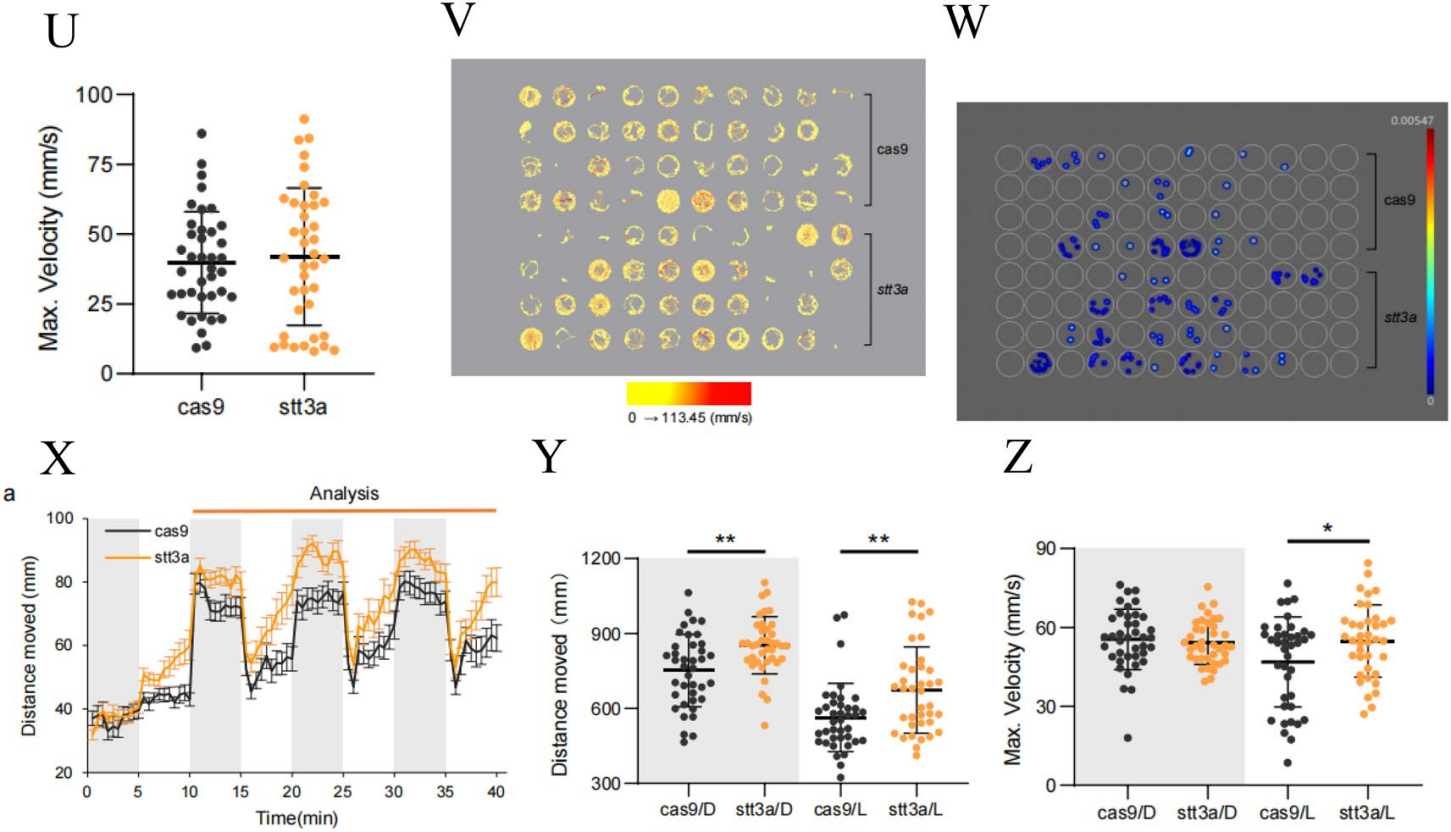
The phenotype validation of CRISPR-Cas9 established heterozygous knockdown zebrafish. **A** Bright-field images of the Cas9 control group and the *stt3a* crispant group zebrafish. **B** Fluorescence images of the brains of the zebrafish in the Cas9 control group and the *stt3a* crispant group. **C**, **D** Measurements of eye distance and body length in the Cas9 control group (*n* = 19 fish) vs. the *stt3a* crispant group (*n* = 29 fish; unpaired t test). **E**–**I** Measurements of the eye distance-to-body length ratio, forebrain area, midbrain area, hindbrain area, and total brain area in the Cas9 control group (*n* = 19 fish) vs. the *stt3a* crispant group (*n* = 29 fish; unpaired t test). **J** Representative fluorescence images of the lower jaw cartilage morphology of the zebrafish in the Cas9 control group and the *stt3a* crispant group. **K**–**I** Measurements of basal length and Ceratohyal angle in the Cas9 control group (*n* = 21 fish) vs. the *stt3a* crispant group (*n* = 20 fish; unpaired t test). **M**–**O** Measurements of Meckel’s length, Palatoquadrant’s length, and Ceratohyal’s length in the Cas9 control group (*n* = 21 fish) vs. the *stt3a* crispant group (*n* = 20 fish; unpaired t test). **P**–**Q** Representative images of zebrafish bone staining in the Cas9 control group and the *stt3a* crispant. Top view and side view. **R** Number of mineralized bones in the Cas9 control group (*n* = 8 fish) vs. the *stt3a* crispant group (*n* = 8 fish; unpaired t test). **S** Plot of the movement distance of the Cas9 control group (*n* = 39 fish) and *stt3a* crispant group (*n* = 39 fish) as a line graph with 1 min intervals; the error bars represent the standard error of the mean (SEM). **T**, **U** Measurements of movement distance and maximum speed in the Cas9 control group (*n* = 39 fish) vs. the *stt3a* crispant group (*n* = 39 fish; unpaired t test). **V** A 15 min trajectory plot where the color of the movement trajectory represents the speed, allowing observation that the Cas9 control group and *stt3a* crispant group exhibit similar trajectory densities and proportions of high-speed movement trajectories. (**W**) When ≥ 40 mm/s is used as the high-speed screening threshold to generate a 15 min heatmap of high-speed movement (with different colors representing frequency values), the proportions of individuals exhibiting high-speed movement and the frequency of high-speed movement occurrence are similar between the Cas9 control group and the *stt3a* crispant group. (**X**) Processing the movement distance data of the Cas9 control group (n = 39) and the *stt3a* crispant group (n = 38) into line graphs with 30 s intervals; the error bars represent the SEM. **Y**, **Z** L means light, D means dark. As the response of zebrafish larvae to changes in light and darkness was not significant in the first cycle, it was not included in the analysis. Data from cycles 2–4 were analyzed by averaging the three points corresponding to each time point in the three cycles. Measurements of movement distance and maximum speed in the Cas9 control group (*n* = 39 fish) vs. the *stt3a* crispant group (*n* = 38 fish; unpaired t test) under light and dark stimuli

**Fig. 5:**
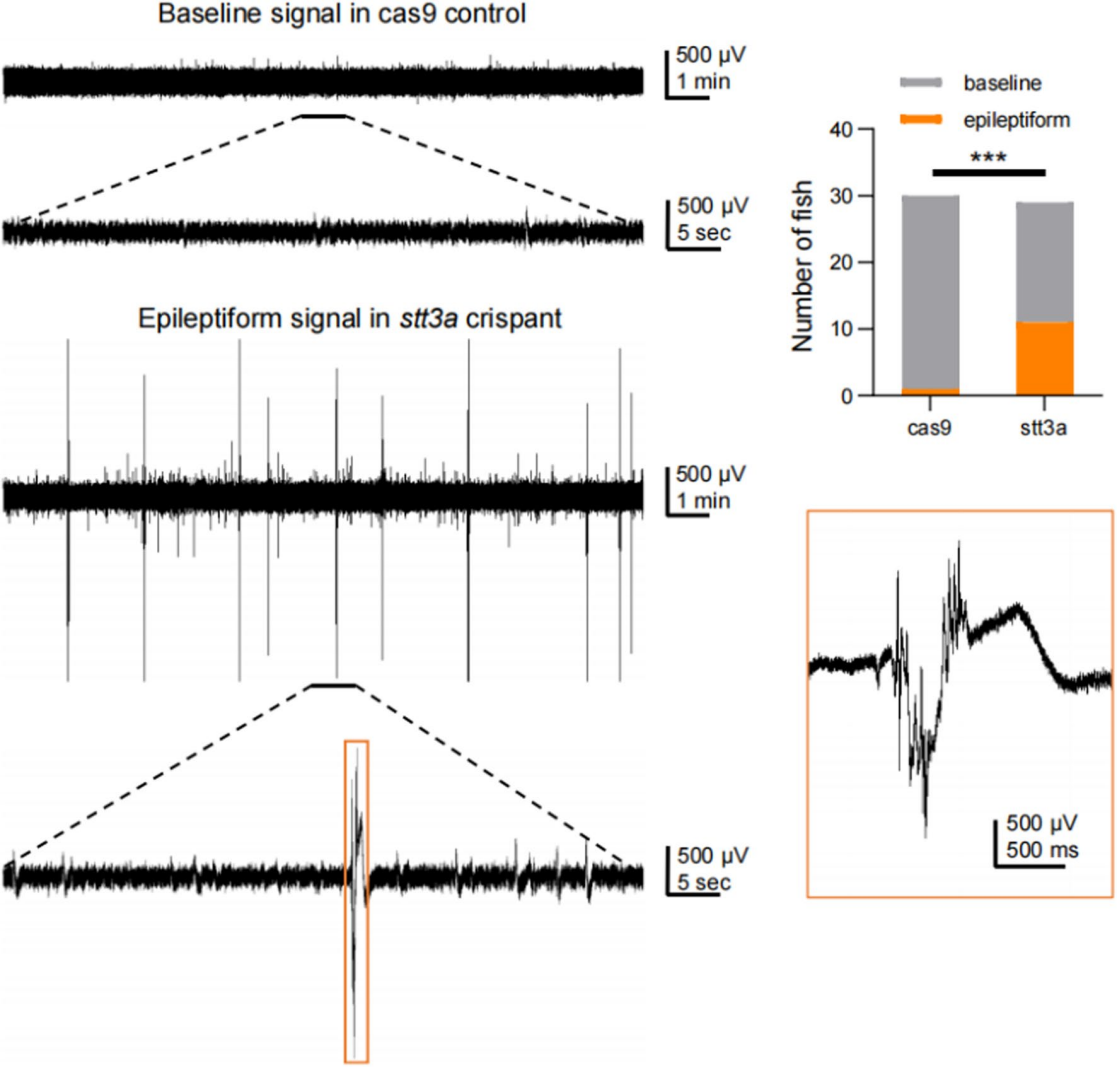
Representative electrophysiological signals of zebrafish in the Cas9 control group and the *stt3a* crispant group. Number of abnormally discharged zebrafish in the Cas9 control group (*n* = 30 fish) vs. *the stt3* crispant group (*n* = 29 fish; chi-square test)

We performed CRISPR-Cas9 genome editing on zebrafish and observed the morphological and neurological phenotypes of F0 zebrafish at 5–9 °C. First, we observed the cranial and body length morphologies of the two groups of zebrafish under a microscope at 5 dpf (Fig. 4 A, B). The results revealed that the *stt3a* crispant group of zebrafish presented significantly increased eye distance (unpaired *t* test, *p* value = 0.0004) and body length (unpaired *t* test, *p* value = 0.0067) (Fig. 4 C, D). However, there were no significant differences between the two groups in terms of the eye distance-to-body length ratio, forebrain area, midbrain area, hindbrain area, or total brain area (Fig. 4E–I). We then observed the morphology of the lower jaw cartilage (Fig. 4J) in the two groups of zebrafish and found that, compared with those of the Cas9 control group zebrafish, the *stt3a* crispant group zebrafish presented a significant increase in Basihyal’s length (unpaired *t* test, *p* value = 0.0014) and Ceratohyal’s angle (unpaired *t* test, *p* value = 0.0445) (Fig. 4 K and l), whereas there were no significant differences in Meckel’s length, Palatoquadrate’s length, or Ceratohyal’s length between the two groups (Fig. 4 M–O). Although the proband did not present with obvious skeletal abnormalities, given that 62.5% of patients in the cases reported by Matthew P. Wilson had skeletal abnormalities [5], we further conducted bone staining in the two groups of zebrafish (Fig. 4 P, Q). The results revealed a significant decrease in the number of mineralized bones in the *stt3a* crispant group of zebrafish compared with the Cas9 control group of zebrafish (unpaired *t* test, *p* value = 0.0471) (Fig. 4R). Behavioral tests were conducted on the two groups of zebrafish. We first examined spontaneous behavior at 1 min intervals and plotted the movement distances of the Cas9 control group and the *stt3a* crispant group as line graphs (Fig. 4S). Statistical analysis was performed on the movement distances and maximum speeds of the two groups, revealing no significant differences in movement distance or maximum speed between the two groups (Fig.4 T, U). Figure 4 V shows the trajectory map for 15 min, with different colors representing varying speeds. The density of movement trajectories and the proportion of high-speed trajectories in the *stt3a* crispant group were similar to those in the Cas9 control group. A high-speed movement heatmap for 15 min was generated using ≥ 40 mm/s as the high-speed screening threshold (different colors represent frequency values), revealing similar proportions and frequencies of high-speed movements in both groups (Fig. 4W). Light (L)-dark (D) stimulation was subsequently applied to the two groups of zebrafish. Movement distance data for the Cas9 control group and the *stt3a* crispant group were processed into line graphs with 30-s intervals (Fig. 4 X). The statistical analysis revealed significant differences in swimming distance between Cas9/D and *stt3a*/D (unpaired t test, *p* value = 0.0010), as well as between Cas9/L and *stt3a*/L (unpaired t test, *p* value = 0.0027) (Fig. 4Y). However, there were no significant differences in maximum speed between Cas9/D and *stt3a*/D (unpaired t test, *p* value = 0.6083), whereas differences between Cas9/L and *stt3a*/L were significant (unpaired t test, *p* value = 0.0277) (Fig. 4Z). Finally, the results of the electrophysiological testing revealed that in the Cas9 control group, 29 out of 30 samples presented normal baseline signals, whereas epileptiform signals were observed in 1 out of 30 samples. In contrast, in the *stt3a* crispant group, 18 out of 29 samples presented normal baseline signals, and epileptiform signals were observed in 11 out of 29 samples (Fig. 5). The difference compared with that in the Cas9 control group was significant (chi-square test, *p* value = 0.0010).

## Discussion

CDG is a hereditary metabolic disorder characterized by systemic involvement. Previously, CDGs, such as *TUSC3*-CDG (MIM: 611,093) [12], *MAGT1*-CDG (MIM: 301,031) [13], *STT3A*-CDG (MIM: 615,596), and *STT3B*-CDG (MIM: 615,597) [14], were considered to be inherited in an autosomal recessive or X-linked manner. However, in recent years, pathogenic heterozygous variants with autosomal recessive inheritance and even dominant inheritance, such as *ALG8*-CDG (MIM: 617,874) [22], *ALG9*-CDG (MIM: 263,210) [23], *DHDDS*-CDG (MIM: 617,836) [24], and *NUS1*-CDG (MIM: 617,831) [25], have been gradually reported. Previously, only autosomal recessive inherited homozygous variants were identified as causing *STT3A*-CDG. However, Matthew P. Wilson et al. [5] reported in 2021 that heterozygous variants at the active site of STT3A could also lead to CDG, suggesting the possibility of autosomal dominant inheritance of *STT3A*-CDG, although in vivo validation is lacking. In this study, we report the first Chinese case of a CDG caused by a *STT3A* heterozygous missense variant, which was also the second study performed after Matthew P. Wilson's study [5], who proposed the potential for autosomal dominant inheritance of *STT3A-*CDG. We subsequently conducted cellular functional validation of this variant and established a heterozygous knockout zebrafish model to validate the phenotype.

The clinical phenotypes of autosomal dominant *STT3A*-CDG and autosomal recessive *STT3A*-CDG appear to not be entirely similar. Previously reported patients with autosomal recessive *STT3A*-CDG mainly presented with developmental delay, microcephaly, failure to thrive, optic atrophy, and epilepsy [6], some of which are not commonly observed in the autosomal dominant form. Our patient experienced only transient feeding difficulties shortly after birth without failure to thrive. Although our patient exhibited distinctive facial features, microcephaly was not present. Additionally, our patient did not experience optic atrophy or seizures, although she had abnormal discharges. Our patient exhibited phenotypic similarities to those reported by Matthew P. Wilson et al., with development delay, distinctive facial features, and short stature. However, unlike that study, where half of the patients (predominantly adults) presented with skeletal dysplasia and muscle spasms, our patient did not present this phenotype. We speculate that this difference may be attributed to our patient being in childhood, or it could be due to phenotypic heterogeneity. However, longer-term follow-up is warranted to elucidate this further.

In the analysis of the trio-WES results of this patient, several factors constitute compelling evidence for identifying the heterozygous variant in *STT3A*. First, the variant was de novo in the patient and was not detected in the normal population. Second, the amino acid affected by this variant is highly conserved across multiple species, and several deleterious prediction software tools have predicted it to be damaged. Additionally, the significant reduction in NK cell CD107a in the patient's peripheral blood, which requires OST-A catalysis [20], further prompts consideration of the pathogenicity of the heterozygous variant in *STT3A*. Finally, our cellular experiments revealed a significant decrease in protein expression caused by the heterozygous variant, suggesting a potential loss of OST-A function. The homozygous missense variants previously identified in *STT3A* (p.Val626Ala and p.Tyr360Ser) are located away from the catalytic site, and their pathogenicity is believed to cause protein instability, leading to reduced protein levels rather than complete loss of catalytic subunit function [14]. However, Matthew P. Wilson suggested that in cases of dominant *STT3A*-CDG, although the mutated protein is stable, those variants are located at the active site of STT3A and are thus likely inactive. Interestingly, the heterozygous missense variant we report here also resides within the catalytic region of STT3A and is near known variants that can affect direct interaction with LLO [5]. Therefore, we speculate that the pathogenic mechanism of this variant may be multifactorial, including unstable protein expression, decreased catalytic activity, and impaired binding to LLO.

To further validate whether heterozygous variants in *STT3A,* which are considered to contribute to the loss of OST-A function, can contribute to the phenotype observed in patients, we constructed a heterozygous knockdown zebrafish model for *stt3a*. We observed phenotypes in the *stt3a* crispant group of zebrafish that resembled those observed in dominantly inherited *STT3A*-CDG patients, including craniofacial dysmorphology (increased eye distance, increased basal length, increased Ceratohyal angle), skeletal abnormalities (reduced number of mineralized bones), ID (reduced adaptability under light‒dark stimuli suggesting abnormal locomotion, orientation, and social behavior), and abnormal charges. Therefore, *stt3a* heterozygously knocked down zebrafish exhibit phenotypes similar to those of patients, providing further clarification that *STT3A* can cause CDG through dominant inheritance.

As mentioned earlier, it was previously believed that pathogenic variants of *STT3A* cause CDG through autosomal recessive inheritance. This may lead clinical physicians to focus more on patients with homozygous variants while overlooking those with heterozygous variants. The new finding that heterozygous pathogenic *STT3A* variation may lead to dominant CDG is highly important for genetic counseling. For the proband we reported in this study, the identification of the pathogenic gene and clinical diagnosis allows the clinical physician to develop further diagnostic and therapeutic plans, as well as prognostic assessments. In the future, if the gene targeted therapies become available, precise targeted treatment can be administered. For the proband's family and the general population, understanding the new genetic pattern aids in developing targeted genetic testing strategies, enabling early identification of individuals carrying pathogenic variants for appropriate medical monitoring and intervention. Additionally, this knowledge helps families make informed decisions regarding reproductive planning, including whether to pursue embryo screening or consider pregnancy termination. Notably, recessive *STT3A*-CDG has been identified in only two families, involving only two distinct pathogenic variants. Dominant *STT3A*-CDGs have also been reported only by Matthew P. Wilson [5] and this study; thus, identifying more affected individuals may expand the phenotypic spectrum.

## Conclusion

In this study, we expanded the phenotype of the dominant *STT3A*-CDG, including developmental delay, distinctive facial features, short stature, and abnormal discharges. The heterozygous variant may be pathogenic through unstable protein expression, decreased enzyme activity, and impaired binding to LLO. Furthermore, we provided in vivo validation through the establishment of a heterozygous knockdown zebrafish model for *stt3a*, further strengthening the compelling evidence for dominant *STT3A*-related pathogenesis.

## Data Availability

The data that support the findings of this study are available from the Children’s Hospital of Chongqing Medical University but restrictions apply to the availability of these data, which were used under license for the current study, and so are not publicly available. Data are however available from the authors upon reasonable request and with permission of the Children’s Hospital of Chongqing Medical University.
